# Engineered CAR-Macrophages as Adoptive Immunotherapies for Solid Tumors

**DOI:** 10.3389/fimmu.2021.783305

**Published:** 2021-11-24

**Authors:** Christopher Sloas, Saar Gill, Michael Klichinsky

**Affiliations:** ^1^ Carisma Therapeutics, Philadelphia, PA, United States; ^2^ Division of Hematology-Oncology, University of Pennsylvania Perelman School of Medicine, Philadelphia, PA, United States

**Keywords:** CAR (chimeric antigen receptor), solid tumor, adoptive cell immunotherapy, synthetic biology, macrophage/monocyte

## Abstract

Cellular immunotherapies represent a promising approach for the treatment of cancer. Engineered adoptive cell therapies redirect and augment a leukocyte’s inherent ability to mount an immune response by introducing novel anti-tumor capabilities and targeting moieties. A prominent example of this approach is the use of T cells engineered to express chimeric antigen receptors (CARs), which have demonstrated significant efficacy against some hematologic malignancies. Despite increasingly sophisticated strategies to harness immune cell function, efficacy against solid tumors has remained elusive for adoptive cell therapies. Amongst cell types used in immunotherapies, however, macrophages have recently emerged as prominent candidates for the treatment of solid tumors. In this review, we discuss the use of monocytes and macrophages as adoptive cell therapies. Macrophages are innate immune cells that are intrinsically equipped with broad therapeutic effector functions, including active trafficking to tumor sites, direct tumor phagocytosis, activation of the tumor microenvironment and professional antigen presentation. We focus on engineering strategies for manipulating macrophages, with a specific focus on CAR macrophages (CAR-M). We highlight CAR design for macrophages, the production of CAR-M for adoptive cell transfer, and clinical considerations for their use in treating solid malignancies. We then outline recent progress and results in applying CAR-M as immunotherapies. The recent development of engineered macrophage-based therapies holds promise as a key weapon in the immune cell therapy armamentarium.

## Introduction

In recent years, cellular immunotherapy has emerged as a promising approach for treating cancer. These therapies harness the immune system’s capacity to clear foreign pathogens and redirect the response towards tumor associated antigens (TAAs). Cells expressing chimeric antigen receptors (CARs) represent a major class of cellular immunotherapy that program immune cells to recognize TAAs and initiate a targeted antitumor response (1). T cells equipped with CAR (CAR-T) have shown clinical efficacy in numerous hematological malignancies, leading to the approval of CD19 and BCMA targeted CAR-T products (2).

Although some hematological malignancies have been readily treated by CAR-T, solid tumors present distinct challenges that limit anti-tumor activity. Unlike hematologic malignancies – which allow for disease access in the peripheral blood, bone marrow, lymph nodes, or spleen – solid tumors require active trafficking, extravasation, and penetration into often immunologically cold and dense fibrotic masses. Developing tumors limit T cell recruitment and infiltration, activate broad suppressive pathways to limit T cell activation, and demonstrate heterogenous TAA expression (3–5). Highlighting the potential of CAR-T against solid tumor targets and the barrier of tumor infiltration, a recent case report demonstrated that anti-HER2 CAR-T were able to clear HER2+ sarcoma that metastasized to the bone marrow – a niche to which CAR-T have access (6). Overwhelmingly, systemic therapy with CAR-T have led to minimal efficacy or transient responses. Numerous efforts have therefore been made to create improved iterations of CAR therapies that overcome solid TME challenges. One approach has been to better equip T cells for the TME using synthetic biology – optimization of CAR framework and signaling domains, deletion of inhibitory receptors with CRISPR, and overexpression of accessory genes such as cytokines, immune ligands, and/or transcription factors (7, 8). Combination therapies with checkpoint inhibitors have also improved CAR-T efficacy, as demonstrated with mesothelin-targeting CAR-T and programmed cell death protein 1 (PD-1) blockade (9).

More recently, significant progress has been made in extending the CAR platform from T cells to alternative leukocytes, such as CAR-expressing NK and gamma-delta (γδ) T cells, whose biological functions may offer improved safety profiles or off-the-shelf potential (10, 11). Compared to conventional CAR-T, these lymphocytes offer reduced risk of alloreactivity, distinct modes of cytotoxicity, and reduced likelihood of cytokine release syndrome (CRS) (11). The success of these novel CAR-lymphocytes raises the question: which immune cells are the best chassis for adoptive CAR immunotherapies? An ideal CAR-immune cell would localize to and persist within the TME while coordinating a broad and robust immune response. The careful choice of immune cell could provide the critical foundation for efficacious CAR therapies, building upon the extensive body of work that has been achieved with CAR-T. Given that CARs have only been tested in a subset of immune cells, continued exploration is warranted to identify the optimum cell type for targeting solid tumors.

Macrophages and other cells of the myeloid lineage could potentially overcome the barriers to treating solid tumors that have hindered CAR-T thus far (12–16). Macrophages are phagocytic cells of the innate immune system that are critical for clearing foreign pathogens (13). Unlike lymphocyte-based therapies, macrophages readily localize to and persist within the TME (14). Macrophages can influence surrounding immune cells in both pro- and anti-inflammatory manners and are adept at remodeling the extracellular matrix (ECM) (13, 15). Macrophages are innate immune cells with potent phagocytic and cytotoxic capabilities that can initiate and potentiate an adaptive immune response *via* T cell recruitment, antigen presentation, co-stimulation, and cytokine secretion (13, 16). Taken together, these effector functions enable epitope spreading and alleviate challenges from target antigen heterogeneity. In this review, we discuss the application of macrophages as cell therapies for targeting solid tumors. We outline strategies and challenges for engineering antitumor functions in adoptively transferred macrophages. We particularly focus on the design of CAR-Macrophages (CAR-M) and provide a current perspective on the field.

## Macrophages for Targeting Solid Tumors

Macrophages are capable of numerous effector functions that could support tumor clearance. Their phenotype is highly plastic and exists across a spectrum of pro- and anti-inflammatory states. Several reviews have comprehensively summarized the dichotomous nature of macrophage polarization (17, 18); here, we provide a brief overview of macrophage phenotype for the context of solid tumor therapies. “Classically activated” (M1) macrophages feature a proinflammatory phenotype that is typically induced by IFN-γ from T helper cells Type 1 (Th1). M1 macrophages secrete pro-inflammatory cytokines such as TNF-α, IL-6, IL-12 and IL-1β which can coordinate an immune response and generate reactive oxygen species to facilitate killing of pathogens (19, 20). Through such mechanisms, M1 macrophages have been shown to exhibit increased tumoricidal activities *in vitro* (21). Activated macrophages upregulate expression of antigen presentation machinery, such as major histocompatibility complex class II (MHC-II), CD80 and CD86, and can thereby serve as antigen presenting cells (APCs) that activate the adaptive immune response by cross-presenting phagocytosed antigens (22–24). Macrophages can thus remove pathogens either directly or by educating the surrounding immune system, both of which would be invaluable for eradicating solid tumors.

In cancer, macrophages often adopt an anti-inflammatory or “alternatively activated” (sometimes referred to as M2) phenotype. Alternatively activated macrophages mediate tissue repair and secrete immunoregulatory cytokines such as IL-4, IL-10, IL-13 and TGF-β, which many solid tumors exploit to support their own growth (25–27). Monocytes are actively recruited to the TME *via* chemoattractants such as CCL2, where they differentiate into tumor-associated macrophages (TAMs) (28). Within the TME, hypoxia and elevated T helper cells Type 2 (Th2) cytokine levels bias TAMs to express tumor-favoring genes (29–31). TAMs support angiogenesis and increased vascular density, thereby promoting tumorigenesis (32). Furthermore, TAMs favor regulatory T cell responses and suppress effector T cell functions through mechanisms including immunosuppressive cytokine secretion, upregulation of programmed death ligand-1 (PDL-1), and enzymatic depletion of L-arginine (33, 34). TAM enrichment in the TME is thus correlated with poor overall prognosis during natural tumor progression (35). While M1 and M2 macrophage categorization is a significant simplification of the intratumoral phenotypic spectrum, macrophages have a dynamic relationship with the TME, supporting the notion that using synthetic biology to control macrophage phenotype and function has significant potential to drive anti-tumor immunity.

## Reprogramming Macrophages for Tumor Suppression With Cellular Engineering

A crucial challenge when generating macrophage-based cancer therapies is enabling proinflammatory effector functions that persist despite the immunosuppressive TME. Efforts to do so broadly fall into two camps – *in situ* reprogramming of TAMs, or *ex vivo* priming of macrophages for adoptive cell transfer. Extensive work has been done on the former to repolarize or deplete TAMs *in situ*, and this work has recently been reviewed elsewhere (36, 37). Here, we focus on *ex vivo* manipulations used in adoptive therapies, including pre-treatment with recombinant proteins, expression of therapeutic transgenes, and gene editing with CRISPR-Cas9.

Historically, adoptive macrophage therapies have used recombinant proteins or small molecules to prime immune responses *ex vivo* (38–42). Earlier studies have shown that IFN-γ treatment enhances macrophage cytotoxicity *in vitro* (21). The first dose-escalation studies in humans therefore isolated peripheral blood monocytes from patients, cultured and differentiated them into macrophages over the course of 7 days, and primed them with IFN-γ for 18 hours prior to infusion (38, 39). However, IFN-γ-primed macrophages had minimal clinical efficacy and failed to induce significant tumoricidal activity. The adoptive transfer of M1-activated macrophages was well-tolerated by patients, with clinical side effects primarily limited to fever and flu-like symptoms (41). Follow-up studies further showed that radiolabeled macrophages were detected at sites of metastasis for more than 7 days following infusion (43). Collectively, these trials demonstrated the feasibility of manufacturing and safety of delivering billions of autologous macrophages through intravenous administration. Results from these early trials thus provided a critical foundation for adoptive myeloid cell therapies.

Recent approaches have used genetic engineering to design macrophages that express proinflammatory transgenes of interest (12, 44–49). These strategies leverage the tumor-homing tendences of macrophages to locally deliver therapeutic cargo and induce cytotoxic activity within the tumor niche. For example, IL-12 is a pro-inflammatory cytokine that activates T and NK cells, but its clinical application is hindered by a narrow therapeutic window that precludes safe systemic administration (50). Multiple groups have attempted to overcome the limitations of IL-12 cytokine therapy by recombinantly expressing the cytokine within genetically engineered macrophages (GEMs) or myeloid cells (GEMys) (44, 45). Preclinical models demonstrated that GEMs and GEMys were able to activate a T cell response and prolong survival without inducing systemic toxicity. Similarly, studies have used GEMs to locally deliver interferon α (IFN-α) or IL-21, which promote immune cell activation, or soluble transforming growth factor receptor II (TGFβR2), which impedes TGFβ-mediated immunosuppression (46, 47). Whereas these approaches stimulate the immune system in a constitutive manner, other studies have focused on confining cytotoxicity to antigen-specific contexts. Gardell et al. engineered antigen-specific killing using GEMs that secrete a bispecific T cell engager (BiTE), which creates a functional bridge between T cell receptors and mutated epidermal growth factor receptor variant III (EGFRvIII) on glioblastoma cells (48). BiTE-secreting GEMs facilitated antigen-specific killing by T cells, which was further augmented by the groups work on IL-12 GEMs (44). Cha et al. similarly targeted EGFR by encoding a secreted single-chain variable fragment (scFv) fused to a Fc moiety, which opsonized tumor cells and induced antibody-dependent cellular phagocytosis (ADCP) by macrophages (49). Notably, engineered macrophages can deliver cargo other than genetically encoded proteins; for example, Huang and colleagues used nanoparticles to engineer macrophages that carry photo-sensitive cytotoxic agents, which are released and induce immunogenic cell death upon exposure to near infrared light (51).

Rather than overexpressing transgenes, inhibiting gene expression using CRISPR-Cas9, zinc finger nucleases, and TALENs have been utilized to augment CAR-T and NK cell function (52–54). Recently, there has been increasing interest in gene editing human myeloid cells, and several nucleofection-based methods for transiently delivering CRISPR-Cas9 ribonucleoproteins (RNPs) to primary myeloid cells have been employed (55, 56), as well as specialized methods using nanoparticles to deliver Cas9 plasmid or RNPs (57, 58). Attractive targets for gene editing include regulatory proteins that block anti-tumor functions, such as signal regulatory protein-α (SIRPα). Cancer cells expressing CD47 stimulate macrophage SIRPα to generate a “don’t eat me” signal to evade phagocytosis (59), and the SIRPα/CD47 signaling axis is now a well-established checkpoint in tumor immunity (60). Ray et al. therefore performed a SIRPα knockout (KO) in the murine monocyte/macrophage cell line RAW264.7 using CRISPR-Cas9 and demonstrated that SIRPα-KO macrophages in this system exhibited enhanced phagocytic ability against cancer cells *in vitro* (58). A subsequent study by Bian et al. demonstrated the therapeutic potential of SIRPα-KO macrophages using syngeneic *in vivo* models and SIRPα-deficient mice (61). The authors in this study demonstrated that SIRPα-deficient macrophages gained potent anti-tumor properties and coordinated a robust immune response when delivered in combination with radiotherapy (61). Similarly promising results were generated by Myers et al. upon targeting the tyrosine phosphatase Shp1, which signals downstream of SIRPα to propagate anti-phagocytic signals (62). Instead of irreversibly editing genes, numerous CRISPR-based technologies regulate gene transcription using a catalytically dead Cas9 (dCas9) and chromatin remodeling factors (63). For example, Liu et al. silenced *CD45*, *CD209* and *TICAM1* genes in primary human monocytes using CRISPR interference (CRISPRi), wherein dCas9 is fused to a KRAB domain (64). Dong et al. used dCas9 fused to a histone methylase to epigenetically silence hypoxia inducible factor 1 subunit alpha (*Hif1α*), which mediates TAM immunosuppressive functions (65). When tested in a murine melanoma model, their *Hif1α* Epigenetically Repressed Macrophage (“HERM”) was able to reprogram the tumor’s immunosuppressive microenvironment and prolong survival (65).

## CAR-M: Macrophages Take the Wheel

**Figure 1:**
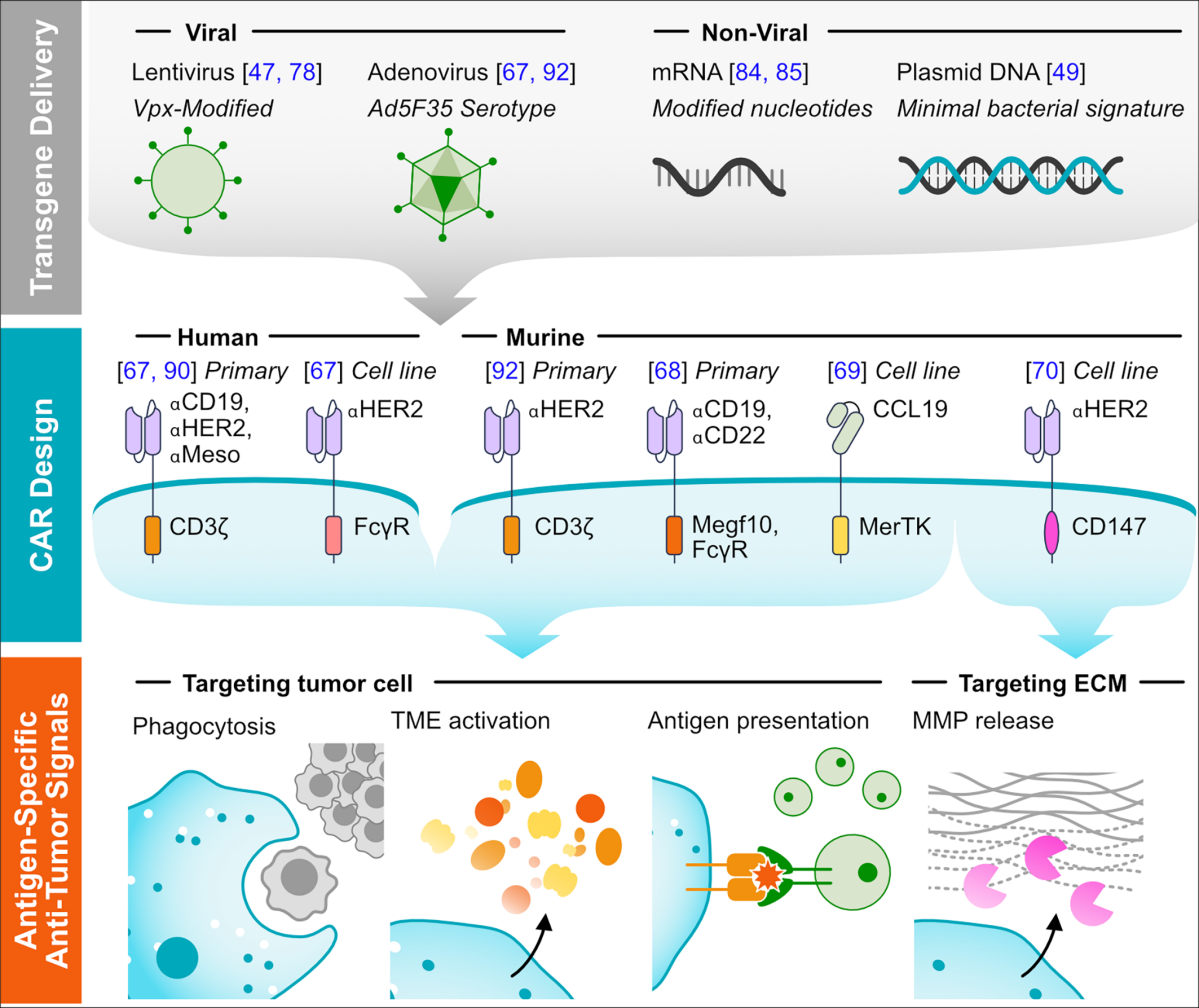
Methods of targeting tumors using CAR-M. (Top) Representative viral and non-viral methods for delivering transgenes to macrophages are listed. (Middle) Representative CAR designs that have been functionally validated in macrophages, with annotated antigen-targeting and cytosolic domains. The system in which the receptors were validated is noted: either human or murine, testing with primary cells or exclusively with immortalized cell lines. (Bottom) Major mechanisms of tumor clearance by CAR-M.

CARs provide a flexible platform for directing immune cell effector functions towards antigen-expressing tumor cells and can promote macrophage antitumor capabilities. Initial studies demonstrating the success translating the synthetic receptors to macrophages are summarized in **Figure 1**.

### Designed, Sealed, and Delivered; Producing CARs for Expression in Macrophages

Current efforts to engineer CAR-M have found that basic CAR design principles from the T cell field hold true for macrophage biology. Traditional CARs are modular transmembrane proteins consisting of an extracellular antigen-recognition domain, a hinge domain, and one or more cytoplasmic signaling domains (1, 66). We have demonstrated that CARs comprising an scFv against broadly representative targets CD19, HER2, and mesothelin, a CD8 hinge and transmembrane domain, and the CD3ζ intracellular domain efficiently redirect macrophages, guiding antigen dependent phagocytosis, cytokine release, and anti-tumor activity (67). Macrophages expressing CARs with CD3ζ, but not with CD3ζ deletions/tyrosine mutations, killed and phagocytosed tumor cells in an antigen-specific manner. Although CD3ζ is canonically used in CARs due to its role in T cell activation, its cytosolic domain bears significant homology with the macrophage-native Fc receptor common gamma chain (FcRγ) that drives ADCP, though with 3 ITAM domains. We confirmed that CAR-M constructed with either the CD3ζ or FcRγ activating domain were functionally similar in phagocytosis assays, conversely complementing earlier findings showing that CD3ζ- and FcRγ-based chimeric receptors were comparably capable of activating T cells (66).

Indeed, the choice of signaling domain is of particular interest when designing CAR-M, and several groups have explored alternative domains. Morrissey et al. designed CAR-M by screening cytoplasmic domains from murine phagocytic receptors including multiple EGF-like-domains protein 10 (Megf10), FcRγ, adhesion G protein-coupled receptor B1 (Bai1) and tyrosine-protein kinase Mer (MerTK) (68). Primary murine macrophages expressing the FcRγ- or Megf10-based CAR exhibited antigen-specific phagocytic capabilities. Niu et al. designed anti-C-C chemokine receptor type 7 (CCR7) CAR-M to target a newly identified LD^hi^CCR7^hi^ immunosuppressive cell population (69). Their design utilized CCL19, the natural ligand of CCR7, as the receptor’s antigen-recognition domain, rather than an scFv. For the intracellular domain, they evaluated activation domains from MerTK, toll-like receptor 2 (TLR2), TLR4, TLR6 and the CAR-T second-generation 4-1BB-CD3ζ. When screened in the RAW264.7 cell line, CAR-M bearing the MerTK activation domain exhibited the greatest tumor cell toxicity. Interestingly, while anti-CCR7 MerTK-based CAR-M performed well in this context, Morrissey et al’s anti-CD19 CAR bearing the same cytosolic domain was unable to bind antigen-functionalized beads, despite expression at the cell surface (68). Such discrepancies hint that optimization and careful functional evaluation is necessary when generating new CAR-M architectures. In a final example, Zhang et al. designed a CAR bearing the activation domain from CD147 (CAR-147), a protein that regulates matrix metalloproteinase (MMP) expression and ECM remodeling (70). Instead of triggering phagocytosis, CAR-147 targeted the tumor ECM by upregulating expression of MMPs upon antigen recognition. While CD147 itself is not macrophage-specific, the utilization of this CAR design allowed for CAR-dependent secretion of MMPs within the tumor. These studies collectively illustrate that the modular CAR template can customize how macrophages respond to target antigens. Future efforts to engineer CAR-M will likely tap into the plethora of sophisticated CAR designs that have been developed for T cells, incorporating tandem activation domains (71), multi-antigen logic gates (72, 73), or drug-sensitive modules (74–76).

Delivering CARs and other transgenes to macrophages can present a challenge for researchers, but recent advances in gene delivery have enabled several viral and non-viral strategies for doing so. Myeloid cells are proficient at detecting and responding to foreign nucleic acids (77), making macrophages and monocytes resistant to genetic manipulation. Bobadilla et al. created novel HIV-1-derived lentiviral particles capable of infecting myeloid cells by leveraging the viral accessory protein Vpx (78). Upon infection, Vpx mediates degradation of SAMHD1, a myeloid-specific HIV-1 restriction factor that inhibits lentiviral transduction by limiting the deoxynucleotide pool and preventing efficient reverse transcription (79). The group demonstrated that modified lentiviral virions containing Vpx can efficiently deliver transgenes to myeloid cells. The Vpx platform can accommodate any pre-existing HIV-based lentiviral vector and thus provides an accessible strategy for modifying myeloid cells (47, 64, 78). Given that macrophages have limited proliferative capacity, we hypothesized that non-integrating, replication deficient adenoviral vectors may allow for efficient and long-term transduction. However, human myeloid cells do not express the Coxackie-adenovirus receptor, which serves as the primary docking site for traditional Ad5 vectors. Monocytes and macrophages highly express CD46, which mediates docking of group B adenoviruses such as Ad35 (80, 81). We thus evaluated the replication-incompetent chimeric adenoviral vector Ad5f35 and demonstrated that Ad5f35 exhibited robust transduction of primary human macrophages and monocytes – with CAR% and viability routinely >80% (67, 82). Ad5f35-transduced macrophages maintained CAR expression for at least 1 month *in vitro* and at least 62 days *in vivo*, as measured by co-expression of CAR-P2A-luciferase. Notably, Ad5f35 activated the macrophage inflammasome and provided a beneficial proinflammatory priming signal, which synergized with CAR activity and rendered the CAR-M locked into an M1 phenotype (83). Such results highlight the prospect of leveraging, rather than evading, the inflammatory response that can occur when delivering genetic material.

Several non-viral strategies have also been developed for engineering monocytes and macrophages. The bacterial origin of plasmid DNA can contribute to inflammation and gene silencing. Plasmids devoid of unmethylated cytosine-phospho-guanine (CpG) dinucleotides – a signature of bacterial DNA – were shown to evade detection by TLR9 and exhibit prolonged gene expression in RAW 264.7 macrophages and primary murine BMDMs (49). Other work has optimized the transient delivery of mRNA to monocytes and macrophages, carefully selecting mRNA modifications and transfection reagents to minimize transfection-induced macrophage toxicity or activation (84, 85). Lastly, transposon systems, which enable non-viral integration into the host genome, have been explored in porcine aortic macrophages (86).

Macrophages may be sourced through several production pipelines. While proof-of-concept studies can be performed in model cell lines such as THP1 and Raw 264.7 or with primary/immortalized murine BMDM, clinical translation necessitates a scalable source of primary human cells. For autologous cell therapies, 2-3x10^9^ peripheral blood monocytes can be obtained by leukapheresis (87), and mobilization with filgrastrim or sargramostim further increases the number of available monocytes by approximately threefold (88). Our CAR-M therapy is manufactured over 1 week using filgrastrim-mobilized CD14^+^ monocytes (67). Monocytes are cultured and differentiated in the presence of granulocyte-macrophage colony-stimulating factor (GM-CSF), which is associated with a pro-inflammatory differentiated phenotype (67, 89). Cells are then transduced with CAR-encoding Ad5f35, which further cements a pro-inflammatory phenotype. To further accelerate manufacturing time, a rapid, same day CAR monocyte process has been developed which yields CAR+ CD14+ monocytes with the capacity to differentiate into M1 CAR-M or CAR-expressing dendritic cells (CAR-DC) (82). Macrophages may be attractive as allogeneic cell therapies since there is no risk of graft *versus* host disease. Immune cells derived from induced pluripotent stem cells (iPSCs) hold potential as a renewable, allogeneic source for CAR-M therapies. Zhang et al. generated iPSC-derived CAR-Macrophages (CAR-iMac) by reprogramming PBMC’s into iPSC’s over the course of several weeks, transducing with CAR-encoding lentivirus, then differentiating into macrophages following a 4-week differentiation process (90). CAR-iMacs were capable of antigen-dependent macrophage functions, such as cytokine secretion and phagocytosis *in vitro*. However, CAR-iMacs differentiated with the current protocol had a lingering anti-inflammatory phenotype, and efficacy was limited when tested in murine models. Additionally in oncology applications, a significant consideration with iPSC-derived CAR-M is MHC-matching; antigen cross-presentation is likely an important component of CAR-M activity downstream of TAA engagement, thus careful study is required to determine whether CAR-M derived from MHC knockout iPSCs can potentiate a sufficient anti-tumor T cell response. Furthermore, continued optimization of the iPSC-to-macrophage differentiation protocol, method of transduction, method of phenotype control, and GMP scale-up are necessary to translate these early findings into the clinic. Provided the process is appropriately scaled, there is theoretically no limit to the number of macrophages that can be expanded from iPSCs or differentiation intermediates, though current optimized protocols yield 2-6×10^7^ macrophage progenitors per harvest (91). Benchmarking iPSC-derived macrophage phenotype against *bona fide* macrophages will be critical for advancing this approach to CAR-M production.

### Mechanisms of Tumor Control by CAR-M

CAR-M therapies are able to clear tumor cells *in vitro* and in preclinical *in vivo* models. *In vitro*, human CAR-M exhibit antigen-specific phagocytosis, cytokine/chemokine secretion, and killing of target antigen expressing targets (67). In two immunodeficient NSGS xenograft models, a single dose of anti-HER2 CAR-M reduced tumor burden and prolonged overall survival against HER2+ SKOV3 tumors. Furthermore, IV-administered CAR-M localized to tumors in several xenograft models and persisted in tumor-free mice (primarily within the liver) for at least 62 days, detected by whole-body bioluminescent imaging of CAR-P2A-luciferase. RNA sequencing revealed that Ad5f35 transduction induced a proinflammatory profile resembling that of classically activated M1 macrophages, which resisted polarization by M2-inducing cytokines *in vitro*. Furthermore, supernatant from CAR-M was sufficient to induce a proinflammatory phenotype in cultured M2 macrophages. These phenotypic results held true in a humanized immune system (HIS) solid tumor xenograft model, where adoptively transferred CAR-M maintained a durable M1 phenotype and induced pro-inflammatory gene expression in host macrophages. *In vitro* analysis further showed that CAR-M could coordinate an antitumor T cell response by recruiting T cells and cross-presenting antigens from phagocytosed cells. Recently, our group established an immunocompetent, syngeneic CAR-M model and demonstrated that murine CAR-M increased intratumoral T cell infiltration, NK cell infiltration, dendritic cell infiltration/activation, and TIL activation (92). We found that CAR-M locally administered in HER2^+^ tumors simultaneously controlled growth of contralateral HER2^-^negative tumors and prevented antigen-negative relapse upon HER2-negative tumor rechallenge, indicating epitope spreading and induction of long-term immune memory. Notably, this work also demonstrated for the first time that CAR-M synergize with PD1 blockade in PD1-monotherapy resistant solid tumor models (92).

Tumor killing by CAR-M was similarly achieved by Niu et al. using CCR7-targeting CAR-M in the RAW264.7 cell line (69). These CAR-M, which exhibited antigen-specific cytotoxicity *in vitro*, prolonged survival and prevented metastasis to distal tissues in a 4T1 breast cancer model. CAR-M recruited CD3^+^ T cells and decreased PD-L1^+^ cells in the tumor site, confirming that engineered macrophages themselves are not the sole driver of the antitumor response. Adoptive macrophage therapy also increased levels of pro-inflammatory cytokines IL1-β, IL-6, and TNF-α in the serum, indicative of a systemic immune response (69).

CAR-M’s ability to facilitate an immune response was underscored by the CAR-147 technology, which targeted the tumor ECM rather than tumor cells directly (70). Zhang and colleagues hypothesized that degrading the dense tumor ECM would improve immune cell infiltration and thereby trigger antitumor activity. CAR-M engineered with a CD147 cytosolic domain upregulated MMP expression in an antigen-specific manner *in vitro*, but exhibited no changes in phagocytosis, killing, or cytokine release. In a HER2^+^ 4T1 breast cancer model, CAR-M slowed tumor growth by reducing its collagen content, enhancing the presence of T cells, and increasing IL-12 and IFN-γ signaling. Taken together, these pioneering studies showcase the ability of CAR-M to infiltrate the tumor niche and initiate a broad anti-tumor response by the host immune system.

## Discussion

### Toward CAR-M Combination Therapies

Co-administration of pharmacological immunotherapies or chemotherapy could further improve CAR-M efficacy. For example, antibody-based immunotherapies rely on macrophage phagocytosis to stimulate an immune response and could be evaluated for augmenting CAR-M efficacy (93, 94). The Fc region of antibodies binds and stimulates macrophage-expressed Fc receptors, leading to ADCP. Antibodies such as trastuzumab and rituximab thus direct macrophages to phagocytose opsonized target cells (95). Antibodies that block phagocytosis-inhibiting signals, such as CD47/SIRPα or the inhibitory Fc receptor FcγRIIB, have enhanced macrophage-mediated immunotherapies (96–98). T cell checkpoint inhibitors blocking PD1 signaling have also been shown to improve macrophage phagocytic capabilities *in vivo* (99). Given the impact of CAR-M on surrounding immune cells, we therefore hypothesized that CAR-M could synergize with PD1 checkpoint inhibitors. In a syngeneic CT26 model, which resists anti-PD1 monotherapy, we demonstrated that the combination of CAR-M with PD1 blockade indeed additively improved overall survival (92). Chemotherapy or radiation therapy could also synergize with CAR-M by inducing immunogenic cell death (100). The efficacy of combining radiation therapy and engineered macrophages was demonstrated by Bian et al. using SIRPα-KO macrophages (61). Furthermore, it is noteworthy that CAR expression is not mutually exclusive from other engineering manipulations described herein. Therefore, future iterations of CAR-M could likely synergize with gene editing or accessory transgene overexpression.

Clinical studies will be crucial to elucidating the toxicity profile of CAR-M in patients. The FDA-approved anti-CD19 CAR-T products tisagenlecleucel, brexucabtagene autoleucel, and axicabtagene ciloleucel carry black box warnings for CRS and neurotoxicity (101). CRS is driven by significant CAR-T expansion and secretion of pro-inflammatory cytokines for sustained periods of time in the peripheral blood. Given that CAR-M have limited expansion potential and do not persist in peripheral blood, severe CRS is not expected, and indeed was not seen in older studies of M1 polarized non-engineered macrophages (41). Engineered macrophages have been shown to persist in pre-clinical glioblastoma models without associated toxicity, indicating that CAR-M may safely interact with the central nervous system (44, 47). A particular concern that may be more relevant for CAR macrophages than CAR T cells is that the TME could subvert tumor-localized CAR-M into a tumor-supporting phenotype (102). Although preclinical models suggest the opposite – that CAR-M reprogram the TME (67) – correlative studies in patients will be necessary to understand the bidirectional dynamics. At present, the first-in-human CAR-M Phase I clinical trial is underway using Carisma Therapeutic’s lead product CT-0508 for treating HER2 overexpressing solid tumors (NCT04660929). Results from this Phase I trial and others will provide invaluable insights to guide the design of safe and effective CAR-M therapies.

### Outlook: Beyond Oncology

Future therapies using engineered macrophages may extend beyond oncology indications. CAR-T have been shown to target fibrotic cardiac and liver tissues, and CAR-M may be even better suited for acellular pathogenic targets (103, 104). Novel therapies could also leverage macrophage tissue remodeling and anti-inflammatory capabilities, rather than their proinflammatory functions. For example, adoptive transfer of anti-inflammatory macrophages has been shown to reduce fibrotic tissue in liver injury models (105). From remodeling synapses to repairing cardiac tissue, macrophages are ubiquitous in maintaining tissue homeostasis, and their therapeutic application should be compatible with myriad tissue contexts (106, 107). In conclusion, macrophage phenotypic plasticity, when combined with synthetic biology, presents an exciting new platform for therapeutic applications to advance cellular engineering and deliver effective immunotherapies.

## Author Contributions

CS wrote the article and designed the figure. SG and MK contributed to writing and critically revised the article. All authors contributed to the article and approved the submitted version.

## Conflict of Interest

CS and MK report being employees of Carisma Therapeutics. MK and SG are co-founders of Carisma Therapeutics. MK and SG hold patents related to CAR-M, which have been licensed to Carisma Therapeutics. SG has received research funding from Carisma Therapeutics.

## Publisher’s Note

All claims expressed in this article are solely those of the authors and do not necessarily represent those of their affiliated organizations, or those of the publisher, the editors and the reviewers. Any product that may be evaluated in this article, or claim that may be made by its manufacturer, is not guaranteed or endorsed by the publisher.

## References

[1] June CH Sadelain M. Chimeric Antigen Receptor Therapy. New Engl J Med (2018) 379:64–73. doi: 10.1056/nejmra1706169 29972754 PMC7433347

[2] Majzner RG Mackall CL. Clinical Lessons Learned From the First Leg of the CAR T Cell Journey. Nat Med (2019) 25:1341–55. doi: 10.1038/s41591-019-0564-6 31501612

[3] Wagner J Wickman E DeRenzo C Gottschalk S. CAR T-Cell Therapy for Solid Tumors: Bright Future or Dark Reality? Mol Ther (2020) 28:2320–39. doi: 10.1016/j.ymthe.2020.09.015 PMC7647674 32979309

[4] Chan JD Lai J Slaney CY Kallies A Beavis PA Darcy PK. Cellular Networks Controlling T Cell Persistence in Adoptive Cell Therapy. Nat Rev Immunol (2021) 22:1–16. doi: 10.1038/s41577-021-00539-6 33879873

[5] Beatty GL O’Hara M. Chimeric Antigen Receptor-Modified T Cells for the Treatment of Solid Tumors: Defining the Challenges and Next Steps. Pharmacol Therapeut (2016) 166:30–9. doi: 10.1016/j.pharmthera.2016.06.010 PMC5035606 27373504

[6] Hegde M Joseph SK Pashankar F DeRenzo C Sanber K Navai S. Tumor Response and Endogenous Immune Reactivity After Administration of HER2 CAR T Cells in a Child With Metastatic Rhabdomyosarcoma. Nat Commun (2020) 11:3549. doi: 10.1038/s41467-020-17175-8 32669548 PMC7363864

[7] Hou AJ Chen LC Chen YY. Navigating CAR-T Cells Through the Solid-Tumour Microenvironment. Nat Rev Drug Discov (2021) 20:531–50. doi: 10.1038/s41573-021-00189-2 33972771

[8] Martinez M Moon EK. CAR T Cells for Solid Tumors: New Strategies for Finding, Infiltrating, and Surviving in the Tumor Microenvironment. Front Immunol (2019) 10:128. doi: 10.3389/fimmu.2019.00128 30804938 PMC6370640

[9] Adusumilli PS Zauderer MG Riviere I Solomon SB Rusch VW O’Cearbhaill RE. A Phase I Trial of Regional Mesothelin-Targeted CAR T-Cell Therapy in Patients With Malignant Pleural Disease, in Combination With the Anti-PD-1 Agent Pembrolizumab. Cancer Discov (2021) 11:2748–63. doi: 10.1158/2159-8290.cd-21-0407 PMC8563385 34266984

[10] Rezvani K Rouce R Liu E Shpall E. Engineering Natural Killer Cells for Cancer Immunotherapy. Mol Ther (2017) 25:1769–81. doi: 10.1016/j.ymthe.2017.06.012 PMC5542803 28668320

[11] Morandi F Yazdanifar M Cocco C Bertaina A Airoldi I. Engineering the Bridge Between Innate and Adaptive Immunity for Cancer Immunotherapy: Focus on γδ T and NK Cells. Cells (2020) 9:1757. doi: 10.3390/cells9081757 PMC7464083 32707982

[12] Biglari A Southgate TD Fairbairn LJ Gilham DE. Human Monocytes Expressing a CEA-Specific Chimeric CD64 Receptor Specifically Target CEA-Expressing Tumour Cells *In Vitro* and *In Vivo* . Gene Ther (2006) 13:602–10. doi: 10.1038/sj.gt.3302706 16397508

[13] Franken L Schiwon M Kurts C. Macrophages: Sentinels and Regulators of the Immune System. Cell Microbiol (2016) 18:475–87. doi: 10.1111/cmi.12580 26880038

[14] Ritchie D Mileshkin L Wall D Bartholeyns J Thompson M Coverdale J. *In Vivo* Tracking of Macrophage Activated Killer Cells to Sites of Metastatic Ovarian Carcinoma. Cancer Immunol Immunother (2006) 56:155. doi: 10.1007/s00262-006-0181-3 16733671 PMC11030026

[15] Yang L Zhang Y. Tumor-Associated Macrophages: From Basic Research to Clinical Application. J Hematol Oncol (2017) 10:58. doi: 10.1186/s13045-017-0430-2 28241846 PMC5329931

[16] Zhu L Hu S Chen Q Zhang H Fu J Zhou Y. Macrophage Contributes to Radiation-Induced Anti-Tumor Abscopal Effect on Transplanted Breast Cancer by HMGB1/TNF-α Signaling Factors. Int J Biol Sci (2021) 17:926–41. doi: 10.7150/ijbs.57445 PMC8040298 33867819

[17] Mosser DM Edwards JP. Exploring the Full Spectrum of Macrophage Activation. Nat Rev Immunol (2008) 8:958–69. doi: 10.1038/nri2448 PMC2724991 19029990

[18] Martinez FO Gordon S. The M1 and M2 Paradigm of Macrophage Activation: Time for Reassessment. F1000prime Rep (2014) 6:13. doi: 10.12703/p6-13 24669294 PMC3944738

[19] Duque GA Descoteaux A. Macrophage Cytokines: Involvement in Immunity and Infectious Diseases. Front Immunol (2014) 5:491. doi: 10.3389/fimmu.2014.00491 25339958 PMC4188125

[20] Fang FC. Antimicrobial Actions of Reactive Oxygen Species. Mbio (2011) 2:e00141–11. doi: 10.1128/mbio.00141-11 PMC3171981 21896680

[21] Le J Prensky W Yip YK Chang Z Hoffman T Stevenson HC. Activation of Human Monocyte Cytotoxicity by Natural and Recombinant Immune Interferon. J Immunol Baltim Md 1950 (1983) 131:2821–6. 6417232

[22] Barrio MM Abes R Colombo M Pizzurro G Boix C Roberti MP. Human Macrophages and Dendritic Cells Can Equally Present MART-1 Antigen to CD8+ T Cells After Phagocytosis of Gamma-Irradiated Melanoma Cells. PloS One (2012) 7:e40311. doi: 10.1371/journal.pone.0040311 22768350 PMC3388056

[23] Tang-Huau T-L Gueguen P Goudot C Durand M Bohec M Baulande S. Human *In Vivo*-Generated Monocyte-Derived Dendritic Cells and Macrophages Cross-Present Antigens Through a Vacuolar Pathway. Nat Commun (2018) 9:2570. doi: 10.1038/s41467-018-04985-0 29967419 PMC6028641

[24] Hume DA. Macrophages as APC and the Dendritic Cell Myth. J Immunol (2008) 181:5829–35. doi: 10.4049/jimmunol.181.9.5829 18941170

[25] Biswas SK Allavena P Mantovani A. Tumor-Associated Macrophages: Functional Diversity, Clinical Significance, and Open Questions. Semin Immunopathol (2013) 35:585–600. doi: 10.1007/s00281-013-0367-7 23657835

[26] Qian B-Z Pollard JW. Macrophage Diversity Enhances Tumor Progression and Metastasis. Cell (2010) 141:39–51. doi: 10.1016/j.cell.2010.03.014 20371344 PMC4994190

[27] Pollard JW. Tumour-Educated Macrophages Promote Tumour Progression and Metastasis. Nat Rev Cancer (2004) 4:71–8. doi: 10.1038/nrc1256 14708027

[28] Qian B-Z Li J Zhang H Kitamura T Zhang J Campion LR. CCL2 Recruits Inflammatory Monocytes to Facilitate Breast-Tumour Metastasis. Nature (2011) 475:222. doi: 10.1038/nature10138 21654748 PMC3208506

[29] Doedens AL Stockmann C Rubinstein MP Liao D Zhang N DeNardo DG. Macrophage Expression of Hypoxia-Inducible Factor-1α Suppresses T-Cell Function and Promotes Tumor Progression. Cancer Res (2010) 70:7465–75. doi: 10.1158/0008-5472.can-10-1439 PMC2948598 20841473

[30] DeNardo DG Barreto JB Andreu P Vasquez L Tawfik D Kolhatkar N. CD4+ T Cells Regulate Pulmonary Metastasis of Mammary Carcinomas by Enhancing Protumor Properties of Macrophages. Cancer Cell (2009) 16:91–102. doi: 10.1016/j.ccr.2009.06.018 19647220 PMC2778576

[31] Casanova-Acebes M Dalla E Leader AM LeBerichel J Nikolic J Morales BM. Tissue-Resident Macrophages Provide a Pro-Tumorigenic Niche to Early NSCLC Cells. Nature (2021) 595:578–84. doi: 10.1038/s41586-021-03651-8 PMC8923521 34135508

[32] Lin EY Li J-F Gnatovskiy L Deng Y Zhu L Grzesik DA. Macrophages Regulate the Angiogenic Switch in a Mouse Model of Breast Cancer. Cancer Res (2006) 66:11238–46. doi: 10.1158/0008-5472.can-06-1278 17114237

[33] Noy R Pollard JW. Tumor-Associated Macrophages: From Mechanisms to Therapy. Immunity (2014) 41:49–61. doi: 10.1016/j.immuni.2014.06.010 25035953 PMC4137410

[34] Noman MZ Desantis G Janji B Hasmim M Karray S Dessen P. PD-L1 Is a Novel Direct Target of HIF-1α, and Its Blockade Under Hypoxia Enhanced MDSC-Mediated T Cell Activation. J Exp Med (2014) 211:781–90. doi: 10.1084/jem.20131916 PMC4010891 24778419

[35] Ryder M Ghossein RA Ricarte-Filho JCM Knauf JA Fagin JA. Increased Density of Tumor-Associated Macrophages Is Associated With Decreased Survival in Advanced Thyroid Cancer. Endocr Relat Cancer (2008) 15:1069–74. doi: 10.1677/erc-08-0036 PMC2648614 18719091

[36] Genard G Lucas S Michiels C. Reprogramming of Tumor-Associated Macrophages With Anticancer Therapies: Radiotherapy *Versus* Chemo- and Immunotherapies. Front Immunol (2017) 8:828. doi: 10.3389/fimmu.2017.00828 28769933 PMC5509958

[37] Jahchan NS Mujal AM Pollack JL Binnewies M Sriram V Reyno L. Tuning the Tumor Myeloid Microenvironment to Fight Cancer. Front Immunol (2019) 10:1611. doi: 10.3389/fimmu.2019.01611 31402908 PMC6673698

[38] Andreesen R Scheibenbogen C Brugger W Krause S Meerpohl H-G Leser H-G. Adoptive Transfer of Tumor Cytotoxic Macrophages Generated in Vitro From Circulating Blood Monocytes: A New Approach to Cancer Immunotherapy. Cancer Res (1990) 50:7450–56. 1701343

[39] Faradji A Bohbot A Schmitt-Goguel M Roeslin N Dumont S Wiesel M-L. Phase I Trial of Intravenous Infusion of Ex-Vivo-Activated Autologous Blood-Derived Macrophages in Patients With Non-Small-Cell Lung Cancer: Toxicity and Immunomodulatory Effects. Cancer Immunol Immunother (1991) 33:319–26. doi: 10.1007/bf01756597 PMC11038571 1651160

[40] Faradji A Bohbot A Frost H Schmitt-Goguel M Siffert JC Dufour P. Phase I Study of Liposomal MTP-PE-Activated Autologous Monocytes Administered Intraperitoneally to Patients With Peritoneal Carcinomatosis. J Clin Oncol (1991) 9:1251–60. doi: 10.1200/jco.1991.9.7.1251 2045866

[41] Andreesen R Hennemann B Krause SW. Adoptive Immunotherapy of Cancer Using Monocyte-Derived Macrophages: Rationale, Current Status, and Perspectives. J Leukoc Biol (1998) 64:419–26. doi: 10.1002/jlb.64.4.419 9766621

[42] Hennemann B Beckmann G Eichelmann A Rehm A Andreesen R. Phase I Trial of Adoptive Immunotherapy of Cancer Patients Using Monocyte-Derived Macrophages Activated With Interferon γ and Lipopolysaccharide. Cancer Immunol Immunother (1997) 45:250–6. doi: 10.1007/pl00006671 PMC11037564 9439648

[43] Hennemann B Scheibenbogen C SchuUmichen C Andreesen R. Intrahepatic Adoptive Immunotherapy With Autologous Tumorcytotoxic Macrophages in Patients With Cancer. J Immunother (1995) 18:19–27. doi: 10.1097/00002371-199507000-00003 8535567

[44] Brempelis KJ Cowan CM Kreuser SA Labadie KP Prieskorn BM Lieberman NAP. Genetically Engineered Macrophages Persist in Solid Tumors and Locally Deliver Therapeutic Proteins to Activate Immune Responses. J Immunother Cancer (2020) 8:e001356. doi: 10.1136/jitc-2020-001356 33115946 PMC7594542

[45] Kaczanowska S Beury DW Gopalan V Tycko AK Qin H Clements ME. Genetically Engineered Myeloid Cells Rebalance the Core Immune Suppression Program in Metastasis. Cell (2021) 184:2033–52. doi: 10.1016/j.cell.2021.02.048 PMC8344805 33765443

[46] Escobar G Barbarossa L Barbiera G Norelli M Genua M Ranghetti A. Interferon Gene Therapy Reprograms the Leukemia Microenvironment Inducing Protective Immunity to Multiple Tumor Antigens. Nat Commun (2018) 9:2896. doi: 10.1038/s41467-018-05315-0 30042420 PMC6057972

[47] Moyes KW Lieberman NAP Kreuser SA Chinn H Winter C Deutsch G. Genetically Engineered Macrophages: A Potential Platform for Cancer Immunotherapy. Hum Gene Ther (2017) 28:200–15. doi: 10.1089/hum.2016.060 27758144

[48] Gardell JL Matsumoto LR Chinn H DeGolier KR Kreuser SA Prieskorn B. Human Macrophages Engineered to Secrete a Bispecific T Cell Engager Support Antigen-Dependent T Cell Responses to Glioblastoma. J Immunother Cancer (2020) 8:e001202. doi: 10.1136/jitc-2020-001202 33122397 PMC7597484

[49] Cha EB Shin KK Seo J Oh D-B. Antibody-Secreting Macrophages Generated Using CpG-Free Plasmid Eliminate Tumor Cells Through Antibody-Dependent Cellular Phagocytosis. Bmb Rep (2020) 53:442–7. doi: 10.5483/bmbrep.2020.53.8.024 PMC7473480 32438971

[50] Berraondo P Etxeberria I Ponz-Sarvise M Melero I. Revisiting Interleukin-12 as a Cancer Immunotherapy Agent. Clin Cancer Res (2018) 24:clincanres.0381.2018. doi: 10.1158/1078-0432.ccr-18-0381 29549160

[51] Huang Y Guan Z Dai X Shen Y Wei Q Ren L. Engineered Macrophages as Near-Infrared Light Activated Drug Vectors for Chemo-Photodynamic Therapy of Primary and Bone Metastatic Breast Cancer. Nat Commun (2021) 12:4310. doi: 10.1038/s41467-021-24564-0 34262026 PMC8280231

[52] Ren J Liu X Fang C Jiang S June CH Zhao Y. Multiplex Genome Editing to Generate Universal CAR T Cells Resistant to PD1 Inhibition. Clin Cancer Res (2017) 23:2255–66. doi: 10.1158/1078-0432.ccr-16-1300 PMC5413401 27815355

[53] Delconte RB Kolesnik TB Dagley LF Rautela J Shi W Putz EM. CIS Is a Potent Checkpoint in NK Cell–Mediated Tumor Immunity. Nat Immunol (2016) 17:816–24. doi: 10.1038/ni.3470 27213690

[54] Daher M Basar R Gokdemir E Baran N Uprety N Cortes AKN. Targeting a Cytokine Checkpoint Enhances the Fitness of Armored Cord Blood CAR-NK Cells. Blood (2020) 137:624–36. doi: 10.1182/blood.2020007748 PMC7869185 32902645

[55] Hiatt J Cavero DA McGregor MJ Zheng W Budzik JM Roth TL. Efficient Generation of Isogenic Primary Human Myeloid Cells Using CRISPR-Cas9 Ribonucleoproteins. Cell Rep (2021) 35:109105. doi: 10.1016/j.celrep.2021.109105 33979618 PMC8188731

[56] Freund EC Lock JY Oh J Maculins T Delamarre L Bohlen CJ. Efficient Gene Knockout in Primary Human and Murine Myeloid Cells by Non-Viral Delivery of CRISPR-Cas9. J Exp Med (2020) 217:1–15. doi: 10.1084/jem.20191692 PMC7336301 32357367

[57] Luo Y-L Xu C-F Li H-J Cao Z-T Liu J Wang J-L. Macrophage-Specific *in Vivo* Gene Editing Using Cationic Lipid-Assisted Polymeric Nanoparticles. ACS Nano (2018) 12:994–1005. doi: 10.1021/acsnano.7b07874 29314827

[58] Ray M Lee Y-W Hardie J Mout R Tonga GY Farkas ME. CRISPRed Macrophages for Cell-Based Cancer Immunotherapy. Bioconjug Chem (2018) 29:445–50. doi: 10.1021/acs.bioconjchem.7b00768 PMC6063311 29298051

[59] Willingham SB Volkmer J-P Gentles AJ Sahoo D Dalerba P Mitra SS. The CD47-Signal Regulatory Protein Alpha (SIRPa) Interaction Is a Therapeutic Target for Human Solid Tumors. Proc Natl Acad Sci (2012) 109:6662–7. doi: 10.1073/pnas.1121623109 PMC3340046 22451913

[60] Barclay AN Berg TKVD. The Interaction Between Signal Regulatory Protein Alpha (Sirpα) and CD47: Structure, Function, and Therapeutic Target. Immunology (2014) 32:25–50. doi: 10.1146/annurev-immunol-032713-120142 24215318

[61] Bian Z Shi L Kidder K Zen K Garnett-Benson C Liu Y. Intratumoral Sirpα-Deficient Macrophages Activate Tumor Antigen-Specific Cytotoxic T Cells Under Radiotherapy. Nat Commun (2021) 12:3229. doi: 10.1038/s41467-021-23442-z 34050181 PMC8163884

[62] Myers DR Abram CL Wildes D Belwafa A Welsh AMN Schulze CJ. Shp1 Loss Enhances Macrophage Effector Function and Promotes Anti-Tumor Immunity. Front Immunol (2020) 11:576310. doi: 10.3389/fimmu.2020.576310 33133093 PMC7550718

[63] Gilbert LA Larson MH Morsut L Liu Z Brar GA Torres SE. CRISPR-Mediated Modular RNA-Guided Regulation of Transcription in Eukaryotes. Cell (2013) 154:442–51. doi: 10.1016/j.cell.2013.06.044 PMC3770145 23849981

[64] Liu B Gentili M Lieb D Eisenhaure T Irvine DJ Hacohen N. An Efficient Lentiviral CRISPRi Approach to Silence Genes in Primary Human Monocytes. Biorxiv (2020) 2020.12.23.424242. doi: 10.1101/2020.12.23.424242

[65] Dong Y Zhang S Gao X Yin D Wang T Li Z. Hif1α Epigenetically Repressed Macrophages *via* CRISPR/Cas9-EZH2 System for Enhanced Cancer Immunotherapy. Bioact Mater (2021) 6:2870–80. doi: 10.1016/j.bioactmat.2021.02.008 PMC7905236 33718668

[66] Eshhar Z Waks T Gross G Schindler DG. Specific Activation and Targeting of Cytotoxic Lymphocytes Through Chimeric Single Chains Consisting of Antibody-Binding Domains and the Gamma or Zeta Subunits of the Immunoglobulin and T-Cell Receptors. Proc Natl Acad Sci (1993) 90:720–4. doi: 10.1073/pnas.90.2.720 PMC45737 8421711

[67] Klichinsky M Ruella M Shestova O Lu XM Best A Zeeman M. Human Chimeric Antigen Receptor Macrophages for Cancer Immunotherapy. Nat Biotechnol (2020) 38:947–53. doi: 10.1038/s41587-020-0462-y PMC7883632 32361713

[68] Morrissey MA Williamson AP Steinbach AM Roberts EW Kern N Headley MB. Chimeric Antigen Receptors That Trigger Phagocytosis. Elife (2018) 7:e36688. doi: 10.7554/elife.36688 29862966 PMC6008046

[69] Niu Z Chen G Chang W Sun P Luo Z Zhang H. Chimeric Antigen Receptor-Modified Macrophages Trigger Systemic Anti-Tumour Immunity. J Pathol (2020) 253:247–57. doi: 10.1002/path.5585 33140856

[70] Zhang W Liu L Su H Liu Q Shen J Dai H. Chimeric Antigen Receptor Macrophage Therapy for Breast Tumours Mediated by Targeting the Tumour Extracellular Matrix. Brit J Cancer (2019) 121:1–9. doi: 10.1038/s41416-019-0578-3 PMC6889154 31570753

[71] Zhao Z Condomines M van der Stegen SJC Perna F Kloss CC Gunset G. Structural Design of Engineered Costimulation Determines Tumor Rejection Kinetics and Persistence of CAR T Cells. Cancer Cell (2015) 28:415–28. doi: 10.1016/j.ccell.2015.09.004 PMC5003056 26461090

[72] Hyrenius-Wittsten A Su Y Park M Garcia JM Alavi J Perry N. SynNotch CAR Circuits Enhance Solid Tumor Recognition and Promote Persistent Antitumor Activity in Mouse Models. Sci Transl Med (2021) 13:eabd8836. doi: 10.1126/scitranslmed.abd8836 33910981 PMC8594452

[73] Dannenfelser R Allen GM VanderSluis B Koegel AK Levinson S Stark SR. Discriminatory Power of Combinatorial Antigen Recognition in Cancer T Cell Therapies. Cell Syst (2020) 11:215–28.e5. doi: 10.1016/j.cels.2020.08.002 32916097 PMC7814417

[74] Wu C-Y Roybal KT Puchner EM Onuffer J Lim WA. Remote Control of Therapeutic T Cells Through a Small Molecule-Gated Chimeric Receptor. Science (2015) 350:aab4077–aab4077. doi: 10.1126/science.aab4077 26405231 PMC4721629

[75] Sakemura R Terakura S Watanabe K Julamanee J Takagi E Miyao K. A Tet-On Inducible System for Controlling CD19-Chimeric Antigen Receptor Expression Upon Drug Administration. Cancer Immunol (2016) 4:658–68. doi: 10.1158/2326-6066.cir-16-0043 27329987

[76] Li H-S Wong NM Tague E Ngo JT Khalil AS Wong WW. Engineering Clinically-Approved Drug Gated CAR Circuits. Biorxiv (2020) 2020.12.14.419812. doi: 10.1101/2020.12.14.419812

[77] Bartok E Hartmann G. Immune Sensing Mechanisms That Discriminate Self From Altered Self and Foreign Nucleic Acids. Immunity (2020) 53:54–77. doi: 10.1016/j.immuni.2020.06.014 32668228 PMC7359798

[78] Bobadilla S Sunseri N Landau NR. Efficient Transduction of Myeloid Cells by an HIV-1-Derived Lentiviral Vector That Packages the Vpx Accessory Protein. Gene Ther (2013) 20:514–20. doi: 10.1038/gt.2012.61 PMC4105013 22895508

[79] Laguette N Sobhian B Casartelli N Ringeard M Chable-Bessia C Ségéral E. SAMHD1 Is the Dendritic- and Myeloid-Cell-Specific HIV-1 Restriction Factor Counteracted by Vpx. Nature (2011) 474:654–7. doi: 10.1038/nature10117 PMC3595993 21613998

[80] Nilsson M Ljungberg J Richter J Kiefer T Magnusson M Lieber A. Development of an Adenoviral Vector System With Adenovirus Serotype 35 Tropism; Efficient Transient Gene Transfer Into Primary Malignant Hematopoietic Cells. J Gene Med (2004) 6:631–41. doi: 10.1002/jgm.543 15170734

[81] Gaggar A Shayakhmetov DM Lieber A. CD46 Is a Cellular Receptor for Group B Adenoviruses. Nat Med (2003) 9:1408–12. doi: 10.1038/nm952 14566335

[82] Gabitova L Menchel B Gabbasov R Pierini S Best A Ross K. Abstract 1530: Anti-HER2 CAR Monocytes Demonstrate Targeted Anti-Tumor Activity and Enable a Single Day Cell Manufacturing Process. Immunology (2021) 81:1530–0. doi: 10.1158/1538-7445.am2021-1530

[83] Lam E Stein S Falck-Pedersen E. Adenovirus Detection by the cGAS/STING/TBK1 DNA Sensing Cascade. J Virol (2014) 88:974–81. doi: 10.1128/jvi.02702-13 PMC3911663 24198409

[84] Moradian H Roch T Lendlein A Gossen M. mRNA Transfection-Induced Activation of Primary Human Monocytes and Macrophages: Dependence on Carrier System and Nucleotide Modification. Sci Rep-UK (2020) 10:4181. doi: 10.1038/s41598-020-60506-4 PMC7060354 32144280

[85] Ohtani Y Ross K Dandekar A Gabbasov R Klichinsky M. 128 Development of an M1-Polarized, Non-Viral Chimeric Antigen Receptor Macrophage (CAR-M) Platform for Cancer Immunotherapy. J Immunother Cancer (2020) 8:A141–1. doi: 10.1136/jitc-2020-sitc2020.0128

[86] Wang X Wang G Wang N Liu J Cai Y Ren M. A Simple and Efficient Method for the Generation of a Porcine Alveolar Macrophage Cell Line for High-Efficiency Porcine Reproductive and Respiratory Syndrome Virus 2 Infection. J Virol Methods (2019) 274:113727. doi: 10.1016/j.jviromet.2019.113727 31493424

[87] Stevenson HC Miller P Akiyama Y Favilla T Beman JA Herberman R. A System for Obtaining Large Numbers of Cryopreserved Human Monocytes Purified by Leukapheresis and Counter-Current Centrifugation Elutriation (CCE). J Immunol Methods (1983) 62:353–63. doi: 10.1016/0022-1759(83)90180-1 6350465

[88] Hennemann B Rehm A Kottke A Meidenbauer N Andreesen R. Adoptive Immunotherapy With Tumor-Cytotoxic Macrophages Derived From Recombinant Human Granulocyte-Macrophage Colony-Stimulating Factor (rhuGM-CSF) Mobilized Peripheral Blood Monocytes. J Immunother (1997) 20:365–71. doi: 10.1097/00002371-199709000-00005 9336743

[89] Fleetwood AJ Lawrence T Hamilton JA Cook AD. Granulocyte-Macrophage Colony-Stimulating Factor (CSF) and Macrophage CSF-Dependent Macrophage Phenotypes Display Differences in Cytokine Profiles and Transcription Factor Activities: Implications for CSF Blockade in Inflammation. J Immunol (2007) 178:5245–52. doi: 10.4049/jimmunol.178.8.5245 17404308

[90] Zhang L Tian L Dai X Yu H Wang J Lei A. Pluripotent Stem Cell-Derived CAR-Macrophage Cells With Antigen-Dependent Anti-Cancer Cell Functions. J Hematol Oncol (2020) 13:153. doi: 10.1186/s13045-020-00983-2 33176869 PMC7656711

[91] Gutbier S Wanke F Dahm N Rümmelin A Zimmermann S Christensen K. Large-Scale Production of Human iPSC-Derived Macrophages for Drug Screening. Int J Mol Sci (2020) 21:4808. doi: 10.3390/ijms21134808 PMC7370446 32645954

[92] Pierini S Gabbasov R Gabitova L Ohtani Y Shestova O Gill S. Abstract 63: Chimeric Antigen Receptor Macrophages (CAR-M) Induce Anti-Tumor Immunity and Synergize With T Cell Checkpoint Inhibitors in Pre-Clinical Solid Tumor Models. Immunology (2021) 81:63–3. doi: 10.1158/1538-7445.am2021-63

[93] Weiskopf K Weissman IL. Macrophages Are Critical Effectors of Antibody Therapies for Cancer. Mabs (2015) 7:303–10. doi: 10.1080/19420862.2015.1011450 PMC4622600 25667985

[94] Bij GJvd Bögels M Otten MA Oosterling SJ Kuppen PJ Meijer S. Experimentally Induced Liver Metastases From Colorectal Cancer can be Prevented by Mononuclear Phagocyte-Mediated Monoclonal Antibody Therapy. J Hepatol (2010) 53:677–85. doi: 10.1016/j.jhep.2010.04.023 20619916

[95] Upton R Banuelos A Feng D Biswas T Kao K McKenna K. Combining CD47 Blockade With Trastuzumab Eliminates HER2-Positive Breast Cancer Cells and Overcomes Trastuzumab Tolerance. Proc Natl Acad Sci (2021) 118:e2026849118. doi: 10.1073/pnas.2026849118 34257155 PMC8307693

[96] Tseng D Volkmer J-P Willingham SB Contreras-Trujillo H Fathman JW Fernhoff NB. Anti-CD47 Antibody–Mediated Phagocytosis of Cancer by Macrophages Primes an Effective Antitumor T-Cell Response. Proc Natl Acad Sci (2013) 110:11103–8. doi: 10.1073/pnas.1305569110 PMC3703977 23690610

[97] Andrechak JC Dooling LJ Discher DE. The Macrophage Checkpoint CD47: Sirpα for Recognition of ‘Self’ Cells: From Clinical Trials of Blocking Antibodies to Mechanobiological Fundamentals. Philos Trans R Soc B (2019) 374:20180217. doi: 10.1098/rstb.2018.0217 PMC6627025 31431181

[98] Roghanian A Teige I Mårtensson L Cox KL Kovacek M Ljungars A. Antagonistic Human Fcγriib (CD32B) Antibodies Have Anti-Tumor Activity and Overcome Resistance to Antibody Therapy *In Vivo* . Cancer Cell (2015) 27:473–88. doi: 10.1016/j.ccell.2015.03.005 25873171

[99] Gordon SR Maute RL Dulken BW Hutter G George BM McCracken MN. PD-1 Expression by Tumour-Associated Macrophages Inhibits Phagocytosis and Tumour Immunity. Nature (2017) 545:495–9. doi: 10.1038/nature22396 PMC5931375 28514441

[100] Emens LA Middleton G. The Interplay of Immunotherapy and Chemotherapy: Harnessing Potential Synergies. Cancer Immunol Res (2015) 3:436–43. doi: 10.1158/2326-6066.cir-15-0064 PMC5012642 25941355

[101] Brudno JN Kochenderfer JN. Recent Advances in CAR T-Cell Toxicity: Mechanisms, Manifestations and Management. Blood Rev (2018) 34:45–55. doi: 10.1016/j.blre.2018.11.002 30528964 PMC6628697

[102] Netea-Maier RT Smit JWA Netea MG. Metabolic Changes in Tumor Cells and Tumor-Associated Macrophages: A Mutual Relationship. Cancer Lett (2018) 413:102–9. doi: 10.1016/j.canlet.2017.10.037 29111350

[103] Amor C Feucht J Leibold J Ho Y-J Zhu C Alonso-Curbelo D. Senolytic CAR T Cells Reverse Senescence-Associated Pathologies. Nature (2020) 583:127–32. doi: 10.1038/s41586-020-2403-9 PMC7583560 32555459

[104] Aghajanian H Kimura T Rurik JG Hancock AS Leibowitz MS Li L. Targeting Cardiac Fibrosis With Engineered T Cells. Nature (2019) 573:430–3. doi: 10.1038/s41586-019-1546-z PMC6752964 31511695

[105] Haideri SS McKinnon AC Taylor AH Kirkwood P Lewis PJS O’Duibhir E. Injection of Embryonic Stem Cell Derived Macrophages Ameliorates Fibrosis in a Murine Model of Liver Injury. NPJ Regener Med (2017) 2:14. doi: 10.1038/s41536-017-0017-0 PMC5677947 29302350

[106] Ding X Wang J Huang M Chen Z Liu J Zhang Q. Loss of Microglial Sirpα Promotes Synaptic Pruning in Preclinical Models of Neurodegeneration. Nat Commun (2021) 12:2030. doi: 10.1038/s41467-021-22301-1 33795678 PMC8016980

[107] Wong NR Mohan J Kopecky BJ Guo S Du L Leid J. Resident Cardiac Macrophages Mediate Adaptive Myocardial Remodeling. Immunity (2021) 54:2072–88. doi: 10.1016/j.immuni.2021.07.003 PMC8446343 34320366

